# Validity and reliability of the FSS in Greek MS patients

**DOI:** 10.1186/2193-1801-2-304

**Published:** 2013-07-05

**Authors:** Daphne Bakalidou, Emmanouil K Skordilis, Sotirios Giannopoulos, Elefterios Stamboulis, Konstantinos Voumvourakis

**Affiliations:** Department of Physiotherapy, TEI of Athens, 4 Apollonos street, N. Makri, 19005 Athens, Greece; Department of Neurology, School of Medicine, University of Ioannina, Ioannina, Greece; Department of Physical Education and Sport Sciences, National and Kapodistrian University of Athens, Athens, Greece; Second Department of Neurology, Attikon Hospital, National and Kapodistrian University of Athens, Athens, Greece

**Keywords:** Factor analysis, Statistical, Fatigue, Multiple sclerosis, Validation studies

## Abstract

**Objectives:**

The study provided validity and reliability evidence of the Fatigue Severity Scale (FSS) in Greek patients with multiple sclerosis (MS).

**Materials and Methods:**

The FSS was administered to 72 MS patients, without co morbid fatigue and 75 matched paired controls with respect to gender and age. Both groups responded to the FSS, SF-36v2, BDI-II and a demographic questionnaire on two time points separated by a 1-week interval. Exploratory and confirmatory factor analysis was performed to test construct validity, concurrent and divergent validity, internal and test-retest reliability were also examined.

**Results:**

Exploratory and confirmatory factor analysis, intercorrelations with BDI-II (*r* = 0.552, *p* < 0.01) and SF-36v2 vitality (*r* = −0.715, *p* < 0.01) and physical functioning (*r* = −0.673, *p* < 0.01) subscales, and differences between patients and non patients (*t*_(145)_ = 6.007*, p* < 0.001), revealed sufficient construct, concurrent and divergent validity evidence. The factor analysis demonstrated a unidimensional structure Cronbach alpha (0.953) and ICC (0.889) was high, indicating that the responses of our sample were internally consistent and stable across time.

**Conclusion:**

The Greek version of FSS is valid and reliable and may be used by clinicians and researchers to assess fatigue of Greek MS patients.

## Introduction

Fatigue is the most common and one of the most disabling symptoms of multiple sclerosis (MS) and has a severe impact on quality of life (Janardhan and Baskhi 2002; Forbes et al. 2006). Fatigue appears also in healthy populations as well as in patients with disease of the Central Neural System (CNS), such as stroke, Parkinson and also in patients with other systemic diseases such as obesity, systemic lupus erythematosus (SLE) and reumatoidis arthritis (RA) (La Chapelle and Finalayson 1998; Friedman and Friedman 1993).

Fatigue related to MS patients has distinctive and unique features (The Canadian MS Research Group 1987; Djaldetti et al. 1996). The most common distinctive feature is significant association with depression (Bakshi et al. 2000a, b; and Pittion- Vouyovitch et al. 2006) its uniqueness stems from the exacerbation by heat (Paty et al. 1998; Krupp et al. 1988). A percentage of 53-85% of MS patients report fatigue (Bakshi et al. 2000a, b; Branas et al. 2000) and the symptoms they experience last for six or more hours daily, often to deteriorate during the afternoon (Krupp et al. 1988). According to Krupp fatigue in multiple sclerosis is “a sense of physical tiredness and lack of energy, distinct from sadness or weakness” (Krupp et al. 1988) and on the above definition the FSS is based. FSS was initially used to assess fatigue among patients with MS and SLE (Krupp et al. 1989).

A number of scales measuring fatigue have been published in the literature, such as the Fatigue Severity Scale FSS (Krupp et al. 1989). Fatigue Impact Scale FIS (Fisk et al. 1994), Modified Fatigue Impact scale MFIS (Multiple Sclerosis Council for Clinical Practice Guidelines 1998), Fatigue Descriptive Scale FDS (Iriate et al. 1999), Fatigue Assessment Instrument FAI (Schwartz et al. 1993), MS-specific FSS MFSS (Krupp et al. 1995), and Daily- Fatigue Impact Scale D-FIS (Benito- Leon et al. 2007). From the above scales the FIS, MFIS, FDS and MFSS are used to measure fatigue only in MS population. The FSS, D-FIS and FAI are used with both MS and other clinical populations. Among them, one of the most widely used scales is the FSS (Krupp et al. 1989), which is a self-administered unidimensional generic 9-item fatigue rating scale. Each item is scored on a 7-point Likert scale from 1 (completely disagree) to 7 (completely agree). The score of FSS is the mean of the nine items, and higher scores indicate worse fatigue. The FSS was developed to measure the modality, severity, frequency and the impact of fatigue in daily functioning and contains items on physical fatigue and social aspects.

The FSS has been translated to numerous languages and its psychometric properties have been assessed in different populations such as patients with chronic hepatitis C (Kleinman et al. 2000), spinal cord injury (Anton et al. 2008), depression (Ferentinos et al. 2010), systematic lupus erythematosus (Mattsson et al. 2008) and the general population (Lerdal et al. 2005). The validity and reliability of the FSS for MS patients has been examined in many countries such as Holland (Rietberg et al. 2010), Turkey (Armutlu et al. 2008), Switzerland (Valko et al. 2008), U.K (Mills et al. 2009), and Iran (Azimian et al. 2009), while it has not been examined in Greece so far. The only two Greek studies found examined patients with major depression (Ferentinos et al. 2010), and Parkinson (Katsarou et al. 2007). Ferentinos et al. (Ferentinos et al. 2010), reported results from exploratory factor analysis, concurrent and discriminant validity, while Katsarou et al. (Katsarou et al. 2007) stated that the FSS had sufficient concurrent validity and internal consistency. There is lack however, of validation in Greek population with MS and no confirmatory factor analytic results have been reported in the literature so far.

Validity and reliability is not a one-time responsibility of test developers, but an ongoing process due to the interaction among participants, context, instrument and purpose of the study and validity evidence cannot be generalized to different situation and populations (Yun and Ulrich 2002; Sherrill and Connor 1999). As reliability and validity of instruments and protocols vary by sample (Thomas and Nelson 2003), administration of a measuring instrument, without validity and reliability evidence may lead to misinterpretation of the findings (Sherrill and Connor 1999). As a consequence, the adaptation and validation of FSS in MS patients would provide clinicians with a valuable assessment tool.

The purpose of the present study was to assess the validity and reliability of the FSS and report results from confirmatory factor analysis, in a sample of Greek MS patients without any other co morbid fatigue-related conditions.

## Methods

The present study examined the construct, concurrent and divergent validity of FSS, in Greek MS patients. Internal consistency and test retest reliability were calculated as well. For the purposes of the study, the scale was into Greek and back translated by another bilingual expert in order to ensure the accuracy of translation. We also used: a) exploratory and confirmatory factor analysis for testing construct validity, b) correlations among fatigue with depressive symptoms and quality of life for testing concurrent validity, and c) differences in fatigue between MS patients and non patients, for testing divergent validity. Cronbach alpha was used for testing internal consistency, while intraclass reliability coefficient was used to assess the stability of the responses. The time interval between the two assessments was 7–10 days, in order to avoid learning effect (Thomas and Nelson 2003).

### Participants

The study was conducted at the Department of Neurology, at the University Hospital ‘Attikon’ of Athens and at the Department of Neurology of the University of Ioannina, School of Medicine in Greece, between October 2010 and May 2011. The research ethics committee of the Attikon University Hospital approved the study’s protocol, and all participants signed and returned an informed consent. All the patients were invited to participate in the study at the outpatient Department of the Neurological clinics. On the visiting day, they filled the questionnaires in a private clinic room, in the presence of the primary researcher who provided explanations, when necessary, to the participants. A total of 72 MS patients were recruited from their respective clinic records. All patients were Greek adults (above 18), with a definite diagnosis of MS, according to the revised McDonald’s criteria (Mc Donald et al. 2001).

Exclusion criteria were as follows in accordance to literature: a) relapse less than one month before the assessment (Armutlu et al. 2008), b)relapse between the two assessments, c) coexisting disease (Rietberg et al. 2010), and d) inability to visit the clinic, follow instructions from the primary researcher and respond to the questionnaires (EDSS ≤ 7.0) (Rietberg et al. 2010).

A group of 75 participants were then randomly selected among visitors of Atticon Hospital. The control group was matched paired according to patient’s sex and age and with no chronic diseases, and no medications for any reason for at least previous month. All participants in both groups responded to the FSS, SF-36v2, BDI-II and a demographic questionnaire on two time points separated by a 1-week interval (Table 1).

**Table 1:** **Demographic characteristics**

Variable	Mean	SD	N
Age			
MS Patients	43.17	10.19	72
Non patients	38.83	10.09	75
Gender			
MS Patients			72
Male			24
Female			48
Non patients			75
Male			24
Female			51
FSS (1^st^ assessment)			
MS Patients	4.41	1.75	72
Non patients	2.89	1.28	75
FSS (2^nd^ assessment)			
MS Patients	4.34	1.80	72
Non patients	2.45	1.17	75
EDSS			
MS Patients	2.40	1.56	72
Vitality			
1^st^ Assessment			
MS Patients	45.67	11.96	72
Control	55.13	07.58	75
2^nd^ Assessment			
MS Patients	45.72	12.14	72
Control	56.17	09.15	75
PF			
1^st^ Assessment			
MS Patients	37.10	13.89	72
Control	52.15	07.60	75
2^nd^ Assessment			
MS Patients	37.84	13.60	72
Control	52.15	07.60	75
BDI-II			
Somatic			
1^st^ Assessment			
MS Patients	09.08	06.27	72
Control	04.96	04.29	75
2^nd^ Assessment			
MS Patients	09.04	06.85	72
Control	04.60	04.12	75
Cognitive			
1^st^ Assessment			
MS Patients	03.88	04.16	72
Control	01.93	02.27	75
2^nd^ Assessment			
MS Patients	03.67	04.58	72
Control	01.53	01.80	75
BDI-II Total			
1^st^ Assessment			
MS Patients	12.96	09.80	72
Control	06.89	05.94	75
2^nd^ Assessment			
MS Patients	12.71	10.94	72
Control	06.13	05.35	75

### Instruments

#### FSS

The fatigue severity scale (FSS) is a nine item self assessment questionnaire (Krupp et al. 1989). Respondents indicate the fatigue they experienced throughout the last two weeks. Permission to use the FSS for the purposes of the present study was obtained by Dr. Krupp. Translation validity evidence of the FSS was then provided through the following steps (Thomas and Nelson 2003; Beaton et al. 2000): a) Forward translation of the FSS in Greek, from a group of 2 medical doctors and 2 Ph.D holders from Universities using English as the primary language. b) Backward translation of the Greek FSS into English, from a second group of 2 medical doctors and 2 Ph.D holders from Universities abroad. c) Accordingly, 5 MS patients and 5 non patients were asked to complete the Greek FSS and identify items requiring modification. The group of patients and non patients indicated that the 9 items of the Greek FSS were accurate and no further linguistic adaptations were required.

#### BDI-II

The Beck Depression Inventory II (BDI-II) (Beck et al. 1996) is a widely used instrument for the detection of existence and severity of depressive symptoms in clinical and general populations. The inventory incorporates 21 items classified under two factors, named cognitive and somatic. The overall Cronbach alpha reported from Beck et al. was .92 while the test retest Pearson r was .93. For the purposes of the study, we used the validated Greek BDI-II version (Tzemos 1984). The use of the BDI-II is for the concurrent validity and in accordance to literature some researchers used it (Armutlu et al. 2008; Katsarou et al. 2007) and others used another depression scale (Ferentinos et al. 2010; Azimian et al. 2009).

#### SF-36v2

The SF-36v2 is a self administered instrument for the assessment of general health status (Ware et al. 1993). For the purposes of the present study, we followed the example of previous researchers (Kleinman et al. 2000; Ferentinos et al. 2010; Mattsson et al. 2008; Azimian et al. 2009; Katsarou et al. 2007) whose studies have shown that FSS is highly correlated to the vitality subscale of SF-36 and used the Vitality and Physical Functioning subscales. Pappa et al. (2005) reported adequate Cronbach alpha coefficient (> 0.70) and construct validity evidence for a normative sample of Greek adults Pappa et al. (2005).

#### EDSS

The Expanded Disability Status Scale (EDSS) (Kurtzke 1983) was used to record disability in the sample of MS patients by a certified clinician.

### Statistical analysis

Exploratory factor analysis was initially used to explore the dimensional structure of the FSS for the Greek sample of MS patients (on patient’s data only) (SPSS). Extraction of factors was carried out with the principal factors method and quartimax rotation was applied. Item loadings above 0.40 were used to retain items under one pre hypothesized factor and testing the dimensionality of the scale. Eigen value above 1.00 (Kaiser’s criterion), the scree plot and percentage of explained variability criteria were used to specify the retained factor. Subsequently, confirmatory factor analysis was used to examine the factorial structure of the FSS, with the EQS software (Bentler and Bonett 1980). Absolute and incremental fit indexes were used to test the sufficiency of the model. The *χ*^2^ and the ratio *χ*^2^/ df were the absolute fit indexes used. The Comparative Fit Index (CFI), Robust CFI and Non-normed Fit Index (NNFI), assessed the model fit as well, since they are considered rather independent from sample size and distribution of scores (Solano-Flores and Nelson- Barber 2001). Indexes range from 0 to 1, and the value ≥ 0.90 represents an acceptable criterion for data fit. Finally, the Standardized Mean Square Residual (SMSR), with the .08 cut-off criterion, examined the residuals.

For testing the concurrent validity hypothesis, Pearson r correlation coefficients were used to examine the relationship between the FSS scores with depressive symptoms (cognitive and somatic) and quality of life (vitality and physical functioning). For the divergent validity, independent samples *t*-test examined the differences between MS patients and non patients in the FSS scores. The 0.05 level of significance was selected to test the above statistical hypotheses. Finally, the Cronbach alpha and intraclass reliability coefficients reported the internal consistency and stability of the scale.

## Results

The scree plot in the exploratory factor analysis revealed a single pre hypothesized factor, with an eigen value of 6.561, explaining 72.902% of the total variance. Moreover, factor loadings were all above the 0.40 cut off criterion.

**Figure 1:**
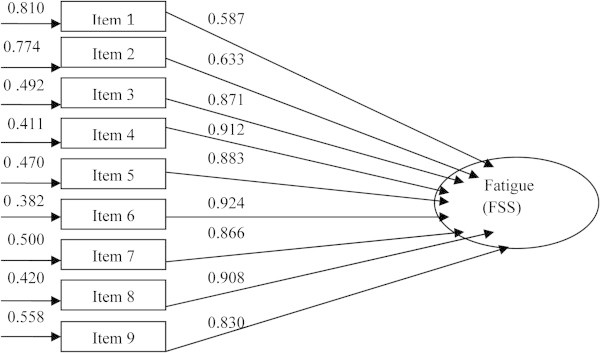
**The 9-item FSS model: item loadings and error variance.**

The distributional properties of the 9 items of the FSS revealed that univariate skewness and kyrtosis indexes and Mardia’s multivariate non normality index of kyrtosis were all at the appropriate range. Based on the above assumptions and the results from the exploratory factor analysis, the data was tested against the pre hypothesized single factor model with 9 items. The confirmatory factor analysis revealed a significant *χ*^2^ value, while the ratio *χ*^2^/ df was at the appropriate range lower than 5 (*x*^2^/ df = 1.958), indicating a proper data fit to the model. Furthermore, the Nonnormed Fit Index (NNFI = 0.974), the Comparative Fit Index (CFI = 0.985) and the Robust CFI (RCFI = 0.993) exceeded the0.90 acceptable criterion of data fit. Finally, the Standardized Root Mean Squared Residual (SRMR = 0.003) was at the appropriate range as well. The overall findings of the confirmatory factor analysis are presented in Figure 1.

Accordingly, the intercorrelations of the FSS scores, during the first assessment, with depressive symptoms (cognitive and somatic) and quality of life (vitality and physical functioning) were examined, for testing the concurrent validity. The intercorrelations were all significant and are presented in Table 2. Finally, the independent samples *t*-test revealed significant differences between MS patients and non patients in the FSS scores (*t*_(145)_ = 6.007*, p <* 0.001). Examination of the mean scores presented in Table 1 revealed that MS patients experienced significantly higher fatigue compared to the control group, confirming therefore the divergent validity hypothesis.

**Table 2:** **Intercorrelations of the FSS scores with depressive symptoms (cognitive and somatic) and quality of life (vitality-VT and physical functioning-PF), during the first assessment**

Variable	FSS	VT	PF	Cognitive	Somatic	BDI-II
FSS	1.00	-0.715**	0.673**	0.456**	0.553**	0.552**
VT		1.00	0.603**	0.590**	0.695**	0.702**
PF			1.00	0.336**	0.448**	0.434**
Cognitive				1.00	0.730**	0.890**
Somatic					1.00	0.961**
BDI-II						1.00

*: Significance at the .05 level.

**: Significance at the .01 level.

Examination of internal consistency of the total sample revealed a Cronbach alpha reliability coefficient of 0.953. The respective coefficients, if item was deleted ranged from 0.942 (item 8) to 0.957 (item 1). Cronbach alpha coefficients, separate for the MS and control groups were 0.961 and 0.912 respectively. The Pearson item total correlation coefficients with the single factor ranged from 0.673 (item 1) to 0.919 (item 8).

Examination of stability for the FSS scores revealed an Intraclass Correlation Coefficient (ICC) of 0.953 for the total sample. The ICC coefficients, separate for the MS and control groups, were 0.881 and 0.792 respectively. Finally, ICCs were used to examine the stability of scores for the SF-36vs and BDI-II subscales. The overall findings are found in Table 3.

**Table 3:** **Reliability analysis**

Variable	Cronbach	ICC
FSS		
Total sample	0.953	0.889
MS Patients	0.961	0.881
Control	0.912	0.792
BDI-II		
Total sample		0.936
MS Patients		0.922
Control		0.938
Cognitive		
Total sample		0.907
MS Patients		0.908
Control		0.852
Somatic		
Total sample		0.930
MS Patients		0.911
Control		0.936
Vitality		
Total sample		0.903
MS Patients		0.891
Control		0.852
Physical functioning		
Total sample		0.980
MS Patients		0.980
Control		0.936

Results from translation, divergent and concurrent validity evidence are presented in Table 4, while a comparative presentation of the Cronbach alpha (a = 0.96) and test retest of Greek MS patients in the present study with those reported from patients in Turkey, Switzerland and Iran is presented in Table 5.

**Table 4:** **Results from translation, divergent and concurrent validity evidence**

Study	Translation	Divergent	Concurrent		
		(patients vs non patients)	FSS & Vitality	FSS & PF	FSS & BDI
MS Patients					
Present study	OK	Significant	-0.72	-0.67	0.55
Armotlu et al.	OK	Significant	-	-	0.43
Azimian et al.	OK	Significant	-0.69	-0.63	-
Valko et al.	OK	Significant	-	-	-
Non MS Patients					
Katsarou et al.	OK	Significant	-0.39	-	0.40
Ferentinos et al. OK	Significant	-0.52	-	-	
Mattsson et al.	OK	Significant	-0.63	-	-
Kleinman et al.	OK	-	-0.76	-	-

**Table 5:** **Cronbach alpha and ICC of MS patients from Greece, Turkey, Switzerland and Iran**

Sample	Cronbach	ICC
Patients from:		
Greece	0.96	0.88
Turkey	0.89	0.81
Switzerland	0.93	-
Iran	0.96	0.93

## Discussion

The present study examined the psychometric properties of the FSS in a Greek sample of MS patients. The results revealed sufficient construct, concurrent and divergent validity evidence. Cronbach alpha and ICC were high, indicating that the responses of our sample were internally consistent and stable across time.

### FSS mean scores, validity and reliability in comparison with MS cohorts across various countries)

The above findings are in agreement with previous studies using the FSS with MS patients from Turkey (Armutlu et al. 2008), Switzerland (Valko et al. 2008), Holland (Rietberg et al. 2010), UK (Mills et al. 2009) and Iran (Azimian et al. 2009) and studies with a variety of populations, such as Parkinson (Katsarou et al. 2007), -SLE (Mattsson et al. 2008), chronic hepatitis C (Kleinman et al. 2000), and depression (Ferentinos et al. 2010). The Cronbach alpha and test retest of Greek MS patients in the present study (a = 0.96) assimilates those reported from patients in Turkey, Switzerland and Iran. Specifically, Armotlu et al. examined the concurrent, divergent validity, internal consistency and stability of the Turkish FSS version and concluded that it was a valid and reliable tool for Turkish MS patients (Armutlu et al. 2008). Valko et al. examined groups of healthy individuals, patients with MS, stroke and sleep disorders from Switzerland and found: a) significant differences among healthy and non healthy patients, suggesting that the FSS is able to discriminate healthy and non healthy groups, and b) high test retest reliability and internal consistency evidence (Valko et al. 2008). Azimian et al. reported convergent validity evidence and sufficient Cronbach alpha and Intraclass coefficients for a sample of MS patients with RRMS from Iran (Azimian et al. 2009). Overall, the present results are in agreement with previous findings and support the psychometric properties of the Greek FSS in MS patients. However, special attention should be paid to items 1 & 2 which exhibit a rather low reliability, an issue already mentioned by Lerdal et al. (2010) who proposed a 7- item scale and Mills et al. who suggested that even a 5-item scale may be of greater validity if items 1,2,6,8 are excluded. (Mills et al. 2009).

Regarding FSS scores, in the present study, the mean FSS score of Greek patients (M = 4.41, SD = 1.75) is comparable to those reported in Switzerland (Valko et al. 2008), (M = 4.66, SD = 1.64), Turkey (Armutlu et al. 2008), (M = 4.81, SD = 1.46) and Iran (Azimian et al. 2009), (M = 5.03, SD = 1.70). These findings indicate a probable universal pattern of fatigue in MS, while methodological issues may account for minor differences between studies.

### FSS validity and reliability in regard to different populations

Regarding different populations, Katsarou et al. reported concurrent and divergent validity evidence, and high internal consistency and stability of the FSS, in Greek patients with Parkinson (Katsarou et al. 2007). Mattsson et al. found sufficient content, construct validity, internal consistency and stability in Sweden, but stated that the sensitivity of the scale must be re examined in the future for patients with SLE (Mattsson et al. 2008). Kleinman et al. reported concurrent validity, internal consistency and test retest reliability evidence for patients with chronic hepatitis C (Kleinman et al. 2000), and finally Feredinos et al. stated that the FSS had satisfactory test retest, internal consistency, construct, concurrent and discriminant validity evidence in a sample of Greek patients with major depression (Ferentinos et al. 2010). The overall results from previous studies with respect to the translation, divergent and concurrent validity evidence are presented in Table 4.

### FSS and other psychometric scales

The FSS exhibited moderate to high correlations with depressive symptoms (cognitive and somatic) and quality of life (vitality-VT and physical functioning-PF), Indeed, fatigue scores show moderate correlations with the severity of depression and the use of FSS as a pure measure of fatigue in depressed patients is under question (Ferentinos et al. 2010). The negative high correlation between FSS and SF-36v2 provide additional evidence for convergent validity with other fatigue scales.

### FSS translation validity

The translation validity process revealed that the nine items were clear and understandable, for all patients and controls who responded twice. The translation validity results were supported from reliability results and the strong psychometric properties of the FSS, emerging through construct, concurrent and divergent validity findings.

### Limitations

There are certain limitations in the present study and the results may not be generalized without caution. First, the number of patients examined, although limited is representative of a wide age spectrum, from 18 to 65 years old. Second, there was no golden standard assessing fatigue in Greek MS patients to compare our findings, third, neither an external validity nor an extended convergent validity by means of other fatigue scales-with the exception of SF-36VIT- had been performed. Finally, responsiveness to clinical changes was not examined and the topic remains open for researchers in the future.

## Conclusions

The present study was the first to report confirmatory factor analytic results, supporting the cross cultural validity of the FSS in Greek MS patients. The Greek version of FSS adapted for MS patients is valid and reliable and it may be used with more confidence in the future to assess fatigue in MS patients from Greece, for clinical and research practice.
